# Buffalo Academy of Medicine

**Published:** 1906-05

**Authors:** Franklin W. Barrows


### Buffalo Academy of Medicine.

*Section of Medicine, April 10, 1906.*

Reported by FRANKLIN W. BARROWS, Secretary.

THE regular meeting of the Section of Medicine was held in the Academy Rooms, Public Library Building, on Tuesday, April 10, 1906, the President of the Academy, Dr. Herbert U.

Williams, presiding, in the absence of the Chairman, Dr. A. W. Bayliss. The Section was called to order at 8:50 p. m.

REPORT OF CASES OF AORTIC STENOSIS

Setting aside the usual order of business the first place was given to DR. NELSON G. RUSSELL, who presented a "Report of Cases of Aortic Stenosis" and exhibited four specimens. He reported six cases of aortic stenosis occurring in about 200 autopsies. Gibson, in his work on the heart, has reported forty cases of pure aortic stenosis and 300 mixed cases; he includes one case in which the aortic second sound was accentuated,—a diagnosis which Dr. Russell is unable to accept. The essayist enumerated the chief signs of aortic stenosis and recounted briefly the histories of the four cases exhibited. One of these cases had been doing fairly well for years, showing very little heart trouble, until the medical attendant began the giving of digitalis to improve the pulse; from this time the case began to do poorly. A more careful study of each individual case would make us more intelligent and cautious in the administering of drugs. In size, the specimens exhibited ranged from nine to thirty-six ounces.

The discussion was opened by DR. DELANCEY ROCHESTER, who called attention to the fact that in the early period of aortic stenosis there is sometimes accentuation of the aortic second sound heard at the junction of the right clavicle and the sternum; when hypertrophy begins to fail or the stenosis becomes more marked, the second sound loses its accentuation. Digitalis is valuable in the first stages of stenosis without marked arterial disease. If the stenosis is moderate, the careful use of digitalis may be very beneficial. While taking this drug the patient should be required to report frequently to his physician.

DR. RUSSELL, in closing the discussion, emphasised again the necessity of a careful study of each case and its lesions in order to avoid mistakes in treatment.

The minutes of the previous meeting were then read and approved.

DR. SIDNEY A. DUNHAM presented a paper entitled:

THE DIAGNOSIS OF INSANITY IN BORDER LINE CASES.

(*Author's Abstract.*)

Strictly speaking, insanity is not a disease of the mind. In mental diseases there is a disturbance of the functions of the brain, or a disease of its structures. The following definition will answer our purpose:—Disease or defect of the brain with a prolonged disturbance of the memory, will, judgment and emotions with false imaginations. We say a prolonged disturbance of the individual's normal standard because there are so many transient morbid states of thought, feeling, and acting that are not insanity. A patient may be legally incompetent and not medically insane; legally insane if he is unable to manage himself and his affairs. A testator is insane if his mind, memory or understanding is unsound.

Hippocrates says "Ira furor brevis est,"—Anger is a brief insanity. The frequent giving away to anger so weakens the control centers that the patient is temporarily unbalanced. Suppressed anger after a time breaks out with greater furor and crime may follow and be committed in a state of temporary insanity. Jealousy in martial relations often leads to insanity. Fear of future happenings or premonitions, so called, may be closely related to insanity. When uncontrolled, anger, jealousy, fear, pride and religious zeal may lead to an unbalanced mind.

MORBID PSYCHICAL STATES NOT WITHIN THE BOUNDS OF PSYCHIATRY.

I will mention several,—toxemia from indigestion or infectious disease, the delirium from alcoholic intoxication and other drug addictions; delirium of fever, injury, shock, fright, epilepsy, apoplexy; the frenzied attacks in hysteria; the depressed or excited states in neurasthenia, the mental confusion in uremic conditions, with or without hallucinations and delirium; also such conditions as narcolepsy, mental preoccupation, trance and somnambulism. Any one of these neurotic states above mentioned, of itself, is not considered insanity. It may develop in any of the above cases where there is a predisposition, such as hereditary defect. In speaking of the normal standard of the individual, every one is a law to himself. Because persons do not think, feel and act the same, it does not follow that they are bordering on insanity. Our normal standard of mental operations depends upon education and training. We don't call the Indian insane because he gets up a war dance to drive out the evil spirits. This has been his training and education. If any one of us doctors should attempt such a procedure, we would be declared insane, because it is a departure from our standard of education.

Believers in witchcraft and Christian science, Dowieism and spiritualism cannot be called insane because they do not believe as we do. When a man of good medical training adopts any of the above, he must be at least on the borderline.

There is no clean cut line between the neuroses and the psychoses; they overlap. The neurologists don't like the word insanity and they wish to cimpromise on the word psychoneuroses. I have not time to mention the different definitions of insanity. But some have gone so far as to attribute any mental change to insanity. If insanity included all mental disturbances, then seven out of ten would be insane but the state would hardly undertake to treat us as such.

If there is no disease of the mind strictly speaking and if in certain cases of insanity and neurasthenia there is no apparent disease of the body, then we are at a loss to name and define the condition. Things that are equal to the same thing are equal to each other, but if we apply mathematics to psychiatry, we are led astray. For example, insanity is a psychosis; neurasthenia or hysteria is a psychosis; therefore, neurasthenia or hysteria is insanity. Insanity must be made to cover more than offensive and dangerous cases, yet the word dangerous is a broad term.

Richard Dewey, of Wisconsin, says that in medicine, law, and common parlance the word insanity is variable and confusing, and suggests that the word psychosis is more indefinite, and would be better to define an indefinite thing. Neurasthenia may be called a psychoneurosis or neuropsychosis, but that will not emphasise anything more definite.

Insanity is nearly always associated with disease of the cortex of the brain. In some severe mental pranks the pathologist finds nothing and we use the term functional. Whenever cerebral nutrition or circulation is disturbed by any toxic condition, the mind is affected and the pathological changes which follow produce peculiar forms of mental disturbances, such as maniacal outbreaks or melancholic depression. Aged people will remember the old and forget the new. Delusions and hallucinations are important in diagnosticating insanity. Those of sight, whether the size of threads or snakes, whether of clouds or landscapes, are of the same importance. Those of hearing, whether of whispers or of words, whether the sounds are pleasing or painful, are of equal importance. Hallucinations are nearly always pathological. The great question to decide is "does the patient believe them as real and do they influence his actions? Real hallucinations do not yield to argument."

We must exclude subjective visual sensations and those of hearing such as make up the aura of an epileptic fit; seeing figures of persons and hearing them talk; also the visual spectra in migraine. We may have the visual sensations without epilepsy or migraine. A case of mine would have attacks of hysteria or extreme nervousness, during which she could see white or black crape on the door. The white would stand for the death of a child, the black for the death of an adult. She would come out of the seance greatly depressed, and would be always sure that some of her near friends had died, as her previous visions had never failed to come true. Also we must exclude volitantes, various floating bodies before the eye, a variety of objects, and the flower gardens seen in simple glaucoma.

We must exclude tinnitis aurium. In tinnitus aurium the sound at first comes apparently from external things, but after a little the patient learns that they are manufactured in the ear. The patient suffering from this disease will frequently ask if someone did not call him or knock at the door, or they may get an idea that they hear thunder. The hissing and rumbling sounds are quite common with these patients. Gowers reports the case of a man who wanted a clock stopped next door and there was not a clock in the house. Many times it is with difficulty that these patients will admit that they are wrong, but that the sound originates in the ear itself. They do not like to admit that their hearing is becoming impaired.

Von Tröltsch reports a case of melancholia with hallucinations of sound in the ear like the crying of a child. By removing the impacted cerumen the sound disappeared; also the melancholia. This seems like a coincidence but the exciting cause is present. In neurotic patients with a tendency to psychical disease these subjective sounds may produce hallucinations and excite mental symptoms.

People are not all insane who have apparent hallucinations and illusions, particularly if they listen to reason, such as seeing their friends and hearing them talk as in spiritualism. When we meet incoherence and erronous conclusions from hallucinations we have insanity to deal with. An illusion is a wrong interpretation, a perverted sensation.

In diagnosticating insanity, very important is a change of sensation. This makes one think the body is changed; for example the hand may seem lost or dead. The patient claims he is petrified by salines, which have been used as enemas. The patient has a pricking sensation and insists that the nurse gave him a hypodermic injection during the night unbeknown to the doctor. When the mind works slowly the patient has an idea that he is under the influence of another, that he is unable to say what he wants to say. A bad feeling in his stomach gives him the idea that he has been poisoned, that some one is using the *x*-ray unbeknown to him.

All perverted sensations may lead to a double personality. Disturbed and uncontrolled emotions and feelings are early manifestations and precursers of insanity. Watch for the shifting glance in mania and the lack of effort in melancholia. * * * * *

Physicians find it much more difficult to differentiate from insanity such conditions as hypochondria, neurasthenia and delirium.

Delirium is a short mental confusion. When prolonged it is called insanity. You will say if it was insanity after two months, it was insanity at first, but we don't diagnosticate other diseases in this way. The indolent ulcer does not become cancer. Congestion of the lungs does not become pneumonia. Congestion of the kidneys is not Bright's disease but may lead to Bright's. Functional troubles, however, do produce diseases. For instance, functional heart troubles associated with violent exercise do lead to vascular changes in the organ itself. Therefore, disturbances of the functions of the brain may continue until the vascular changes are so pronounced as to develop a real disease.

### NEURASTHENIA.

This is a term not satisfactory to doctor or patient, but there is none better. There are times when the neurasthenic borders on insanity. There is a nerve weakness or disturbance sufficiently morbid to be classed in some way. It is a good term when there is not much the matter, but the patient feels so bad that he is sure he has something the matter and wants a name for it. The sensations are painful. There is hyperasthenia of special senses; also muscæ volitantes, and mental and physical fatigue; patient talks in low voice; memory is poor; colects thoughts with difficulty. The eye easily tires; the ear is unduly sensitive to noise; the effort of speech is tiresome; the social duties are burdensome. The patient admits that he is no longer able to pursue his usual work. By proper rest and food and environment we can cure these cases before the psychical symptoms develop.

It is only a step from severe neurasthenia to mental disease. There is the same disturbed psychical functions, the same nutritive changes in the nerve cells, the same class of symptoms only in an exaggerated degree. It is not long before some hallucinations of the senses occur. The pimple on the face is so magnified and so disturbs the individual for fear it may become cancer, that she refuses to see her friends and isolates herself in her own room and, later, will attempt suicide rather than endure the trouble any longer. Another patient will soon imagine that he is losing his business; that his neighbor is undermining his affairs by talking about him; that some one is following him daily, and if not advised and treated will soon report to the police about his imaginary enemies or write them threatening letters.

When the failing memory of the neurasthenic becomes imaginary and beyond control, when the weak mind becomes exalted or greatly depressed and the will power weakens and the emotions are uncontrolled, then we have passed from the neurotic side over the line into the psychic phenomena.

### HYPOCHONDRIA.

Symptoms are self-centered, there is a distress of mind, the bowels don't move, the kidneys are paralysed, the food don't digest and these persons continually appeal for relief. The breathing is imperfect, the heart don't beat right. Not only are his sensations misleading, but he loses control of the emotions. He desires to excite sympathy and threatens suicide.

While many hypochondriacs are insane in many ways, they cannot be treated as such unless there are other proofs of insanity. The term is applied to men in contradistinction to hysteria, which is applied to women. They have great mental depression over slight bodily ills, sometimes imaginary diseases. They are over anxious about themselves. Small ailments are exaggerated. They are continually watching themselves. One patient believes he has cancer or consumption, or heart disease or is losing his mind. He is different from the insane individual who believes he has become filled up so he cannot swallow, or that his head is full of wheels, or that his neck has become dislocated, or refuses to eat because he cannot pay for it; that he has committed the unpardonable sin; that he is being punished for some sin committed in early life.

Some forms of paranoia are difficult to diagnosticate. The assassins of the world are of the paranoic class, being congenitally defective peculiar from childhood, and have frequent attacks of mental depression. When such a person's vitality is reduced, he becomes suspicious, has ideas of conspiracy, is deprived of business rights and property, and is kept down by his enemies. At his best he sees great opportunities openiing up before him, he has great things right in his clutches. In some experience or casual remark by a friend, or quotation from the newspaper, he finds a key to his past career. He swears because he reads in the paper that Lincoln swore, has cards printed with "Honorable" on them, wants to be president; purchases a revolver to be ready for any attack; walks down Main street without overcoat to show his frame and powerful physique. To improve the dents or dimples in his face he wants parafine injected. He cannot always be treated as insane because he has lucid intervals.

From a medical and legal standpoint, socially and politically this is an important subject. Borderline cases do not stand still. There is a tendency to return to normal or get worse. Nervous symptoms decrease and mental symptoms increase. We are often deceived by the upward or downward turns of the neuron. The patient gains in weight and nerve energy and becomes quite normal; then, without any known cause, relapses occur and he goes down into the depths of mental exhaustion greater than at first. It is during this good term that their friends take them home from the asylum or sanitarium and during this downward turn that they are not properly watched and crime and suicide is the result.

Ordinarily it is easy to diagnosticate insanity, but there are many cases in which it is difficult. The various toxemias, without treatment, often develop into some form of insanity. In nervous cases when they form the drug habit we notice that there is a steady progress toward mental disturbances. Where there is a hereditary tendency or neurotic diathesis, previous attacks of insanity, drug addiction, unstable nerves,—as chorea, hysteria, epilepsy,—we only need some exciting cause, such as fright, injury, loss of friends, loss of money to light up a prepared brain. The patient who was devout may suddenly become profane; the social, unsocial; the mild tempered, irritable; the industrious, indolent; the temperate, inclined to dissipate; the cheerful, sullen; the conservative business man becomes a speculator and goes to the wall and resorts to suicide to drown the ills he has and flees to those he knows not of.

DISCUSSION.

Dr. Henry P. Frost opened the discussion. In diagnosticating insanity we cannot demand any definite and pathognomonic signs as we do in diagnosticating other diseases. It is important, however, to know the signs of the early stage of general paresis, in order that we may commit the patient against his will. Dr. Meyer thinks that early diagnosis in from five to ten per cent. of paretics is impossible. In these cases lumbar puncture offers considerable hope of success. An unusual number of lymphocytes in the spinal fluid obtained by this means is confirmatory evidence of other signs of possible paresis. The department of lunacy in this state, and elsewhere as well, wishes to get hold of these incipient cases of general paresis as early as possible in order to give them a chance of improvement. Physicians should not be in too great a hurry to commit delirious cases. The delirium may be the accompaniment of acute infection, or of some other disease not suited to the purposes of the State Hospital. As a general thing, physicians are apt to commit cases to the asylum too early rather than too late.

Dr. J. D. Bonnar thought that in dealing with these borderline cases it is important to differentiate the functional and the structural diseases. Many cases of so-called insanity, probably many of the cases ending in suicide, are the result of temporary passion. The authorities should cut off all supplies of poisonous drugs from such cases, as it is proposed to do in our city. Neuresthenia he regards, not as a disease, but as the result,—the symptom,— of a disease.

Dr. Dunham, in closing the discussion, wished it understood that he believes neurasthenia is a severe functional disease, doing well away from home and poorly under home influences. The friends of such patients are frequently responsible for a great deal of unconscious nagging. This is proven by the fact that a very large proportion of suicides occur at home.

The Section adjourned at 10.20. Attendance 30.

